# Coleman County Medical Society

**Published:** 1904-02

**Authors:** 


### Coleman County Medical Society.

At the last meeting of this society the following officers were elected: Dr. C. M. Alexander, President; Dr. J. D. McCann, Vice-President; Dr. T. M. Hays, Secretary and Treasurer; Dr. Newton Long, delegate to State convention. The Board of Censors is constituted of Drs. G. B. Beaumont, J. M. Mathews and C. M. Alexander.

